# A Rising Crisis: Escalating Burden of Diabetes Mellitus and Hypertension‐Related Mortality Trends in the United States, 2000–2023

**DOI:** 10.1002/clc.70167

**Published:** 2025-07-02

**Authors:** Hibah Siddiqui, Zahra Imran, Dua Ali, Maryam Sajid, Taimor Mohammed Khan, Hussain Salim, Muhammad Salik Uddin, Shaheer Qureshi, Muzammil Farhan, Saad Ahmed Waqas

**Affiliations:** ^1^ Department of Medicine Dow University of Health Sciences Karachi Pakistan; ^2^ Imperial College London UK

**Keywords:** CDC‐WONDER, Diabetes Mellitus, hypertension, mortality, trends

## Abstract

**Introduction:**

Diabetes mellitus and hypertension are major contributors to cardiovascular and renal disease mortality, yet their combined long‐term impact on mortality trends in the United States remains underexplored. This study evaluates national trends in DM and hypertension‐related mortality from 2000 to 2023, analyzing disparities across sex, age groups, race/ethnicity, urbanization, and geographic regions.

**Methods:**

We analyzed mortality data from the CDC‐WONDER database, identifying deaths with DM and hypertension as listed causes among adults aged 25 and older. Age‐adjusted mortality rates (AAMRs) per 100,000 were calculated, and temporal trends were assessed using Joinpoint regression to determine annual percentage changes.

**Results:**

A total of 2,742,668 DM and hypertension‐related deaths were recorded. The AAMR nearly doubled from 33.7 per 100,000 in 2000 to 66.2 per 100,000 in 2023. A sharp increase was observed from 2018 to 2021 (APC: 16.3 [95% CI: 11.8–19.6]), followed by a decline through 2023. Men had consistently higher mortality rates than females. Mortality rates were highest among older adults (65+ years), Non‐Hispanic Black individuals, and nonmetropolitan populations. The South had the highest mortality rates, with Mississippi and the District of Columbia reporting the greatest burden.

**Conclusions:**

DM and hypertension‐related mortality has significantly increased over the past two decades, with notable demographic and geographic disparities. Public health interventions should prioritize high‐risk populations to mitigate mortality trends and improve health equity.

## Graphical abstract

Diabetes mellitus and hypertension‐related mortality nearly doubled in the United States from 2000 to 2023, with striking disparities across sex, age, race, and geography. Men, Non‐Hispanic Blacks, older adults, and residents of the South experienced the highest mortality rates, underscoring the urgent need for targeted strategies to reduce this growing burden.

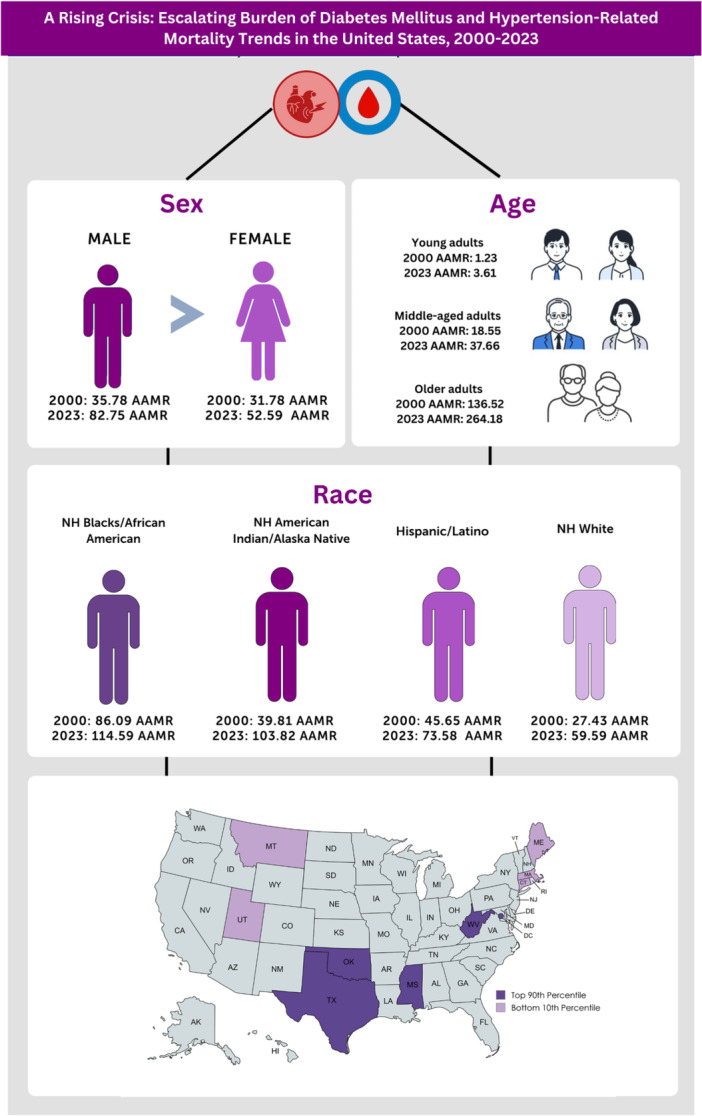

## 1 Introduction

The global incidence of diabetes mellitus (DM) has increased approximately fourfold in recent decades [1]. In the United States, between 2019 and 2022, approximately 4 out of every 1000 youths and 5 out of every 1000 adults were diagnosed with type 1 diabetes [2]. DM is a significant contributor to the development of multiple chronic diseases [3]. Similarly, hypertension affects nearly half of the adult population in the United States [4]. In 2019, the estimated annual economic burden of high blood pressure in the U.S. was approximately $219 billion [5]. Notably, individuals with DM are twice as likely to develop hypertension compared to those without the condition [6]. Furthermore, DM is more frequently observed in individuals with hypertension than in the general population, highlighting a bidirectional and interdependent relationship between the two diseases [7].

The coexistence of DM and hypertension may be attributed to shared underlying risk factors, including metabolic dysfunction and environmental influences. The pathophysiology of hypertension in DM involves maladaptive physiological changes, including dysregulation of the autonomic nervous system, immune system alterations, heightened activation of the renin‐angiotensin‐aldosterone system (RAAS), and adverse environmental exposure [8]. Hypertension is present in 50% to 80% of individuals with type 2 diabetes—who comprise over 90% of the DM‐affected population—compared to approximately 30% of those with type 1 diabetes [9, 10]. Furthermore, individuals diagnosed with hypertension at the onset of DM experience higher all‐cause mortality rates compared to those with DM but normal blood pressure [11].

It is predicted that by 2045, approximately 1 in 8 adults—equivalent to 783 million individuals—will be living with DM, representing a 46% increase from current levels [12]. Given the mounting prevalence of DM and its profound impact on the vascular system, elucidating the intricate relationship between DM and hypertension is of paramount importance. However, the present burden and trends in DM and hypertension‐related mortality remain unexplored. This review analyzes CDC‐WONDER data from 1999 to 2023 to provide a comprehensive overview of demographic, regional, and temporal trends in DM‐hypertension comorbidity and mortality, with a particular focus on identifying high‐risk populations and disparities by race, sex, and geographic location.

## 2 Methods

### 2.1 Study Setting and Population

We analyzed mortality data related to DM and hypertension obtained from the CDC‐WONDER database, which contains death certificate data from all fifty states and the District of Columbia [13]. We used the Multiple Cause of Death Public Use Record to retrieve data about patients who died with both DM and hypertension as either an underlying cause or contributing cause of death in the United States from 1999 to 2023. We collected death records for patients aged 25 and older using the following International Classification of Diseases, 10th Revision, Clinical Modification codes: E10‐E14 for DM AND I10‐I15 for hypertension. These codes have been used by other researchers to identify these conditions in this database [14, 15, 16]. Additionally, we followed the guidelines established by the reporting standards of the Strengthening the Reporting of Observational Studies in Epidemiology (STROBE) [17]. The study did not require approval from the local institutional review board because it used an anonymous public data set provided by the government.

### 2.2 Data Extraction

The extracted data included population, year, and demographics such as sex, race/ethnicity, age, and regional details. Race/ethnicity was classified into the following categories: Hispanic or Latino, Non‐Hispanic (NH) Black or African American, NH White, and NH American Indian or Alaska Native. The Urban–Rural classification was based on the National Center for Health Statistics Urban–Rural Classification Scheme. The population was divided into urban (large metropolitan area, medium/small metropolitan area) and rural (population < 50,000) counties according to the 2013 U.S. census classification [18]. Regions were classified as Northeast, Midwest, South, and West based on U.S. Census Bureau definitions [19].

### 2.3 Statistical Analysis

The age‐adjusted mortality rates (AAMRs) per 100,000 individuals from 1999 to 2023 were calculated to analyze the trends in mortality related to DM and Hypertension. AAMRs were calculated by standardizing DM and hypertension‐related deaths to the 2000 U.S. population, with 95% confidence intervals [20]. The AAMRs were used to analyze mortality patterns across various demographic classifications. The trends in AAMR were determined using the Joinpoint Regression Program (Joinpoint Version 5.1.0, National Cancer Institute) that reported annual percentage change (APC) along with 95% CI. The APCs were categorized as either increasing or decreasing based on their statistical significance, with a P‐value threshold set at < 0.05. Additionally, sensitivity analyses were conducted by considering cases where hypertension was the underlying cause of death and DM was a multiple cause of death.

## 3 Results

From 2000 to 2023, there were a total of 2,742,668 deaths with hypertension and DM listed as a cause of death **(**Table 1, Table S1 **)**. Information on the location of death was available for 2,183,018 deaths. Of these, 40.1% occurred in medical facilities, 20.3% occurred in nursing homes/long‐term care facilities, 3.0% occurred in hospices, and 32.4% at home **(**Table S2, Table S3 **)**.

**Table 1:** Frequency and age‐adjusted rates per 100,000 adults with diabetes mellitus and hypertension concomitantly stratified by sex, race, census region, and location of death.

	Deaths	Population	AAMR 2000 (95% CI)	AAMR 2023 (95% CI)	AAPC (95% CI)
**Overall**	2,742,668	4,982,722,493	33.67 (33.40–33.94)	66.23 (65.92–66.54)	2.93 (2.43 to 3.69)
**Sex**					
**Men**	1,419,324	2,068,596,622	35.78 (35.34–36.22)	82.75 (82.23–83.27)	3.65 (3.15–4.47)
**Women**	1,323,344	2,225,435,144	31.78 (31.44–32.12)	52.59 (52.23–52.96)	2.17 (1.69–2.86)
**Non‐Hispanic race**					
**NH White**	1,814,571	3,393,977,066	27.43 (27.17–27.70)	59.59 (59.25–59.93)	3.40 (3.06–3.89)
**NH Black/African American**	517,091	582,396,578	86.09 (84.59–87.58)	114.59 (113.29–115.90)	0.75 (0.16–1.31)
**NH American Indian/Alaska Native**	25,030	36,666,308	39.81 (35.29–44.33	103.82 (98.89–108.75)	3.05 (2.33–3.73)
**Hispanic race**	279,965	684,525,097	45.65 (44.20–47.10)	73.58 (72.55–74.62)	1.43 (0.64–2.32)
**Census region**					
**Northeast**	425,853	912,592,124	29.67 (29.12–30.23)	48.09 (47.49–48.7)	1.79 (1.26–2.47)
**Midwest**	587,365	1,069,705,126	33.40 (32.85–33.95)	60.57 (59.93–61.21)	2.58 (2.12 to 3.18)
**South**	1,139,817	1,852,271,479	36.26 (35.79–36.73)	79.74 (79.19–80.29)	3.00 (2.57–3.37)
**West**	589,633	1,148,153,764	33.61 (33.01–34.21)	63.60 (62.97–64.23)	2.76 (2.34–3.41)
**Location of death**					
**Medical facility**	875,075	•	•	•	•
**Hospice facility**	66,441	•	•	•	•
**Nursing home/long term care**	442,957	•	•	•	•
**Decedent's home**	707,593	•	•	•	•

• Represents data that was unavailable from CDC WONDER.

### 3.1 Annual Trends for Hypertension and DM Comorbidity Mortality

**Figure 1:**
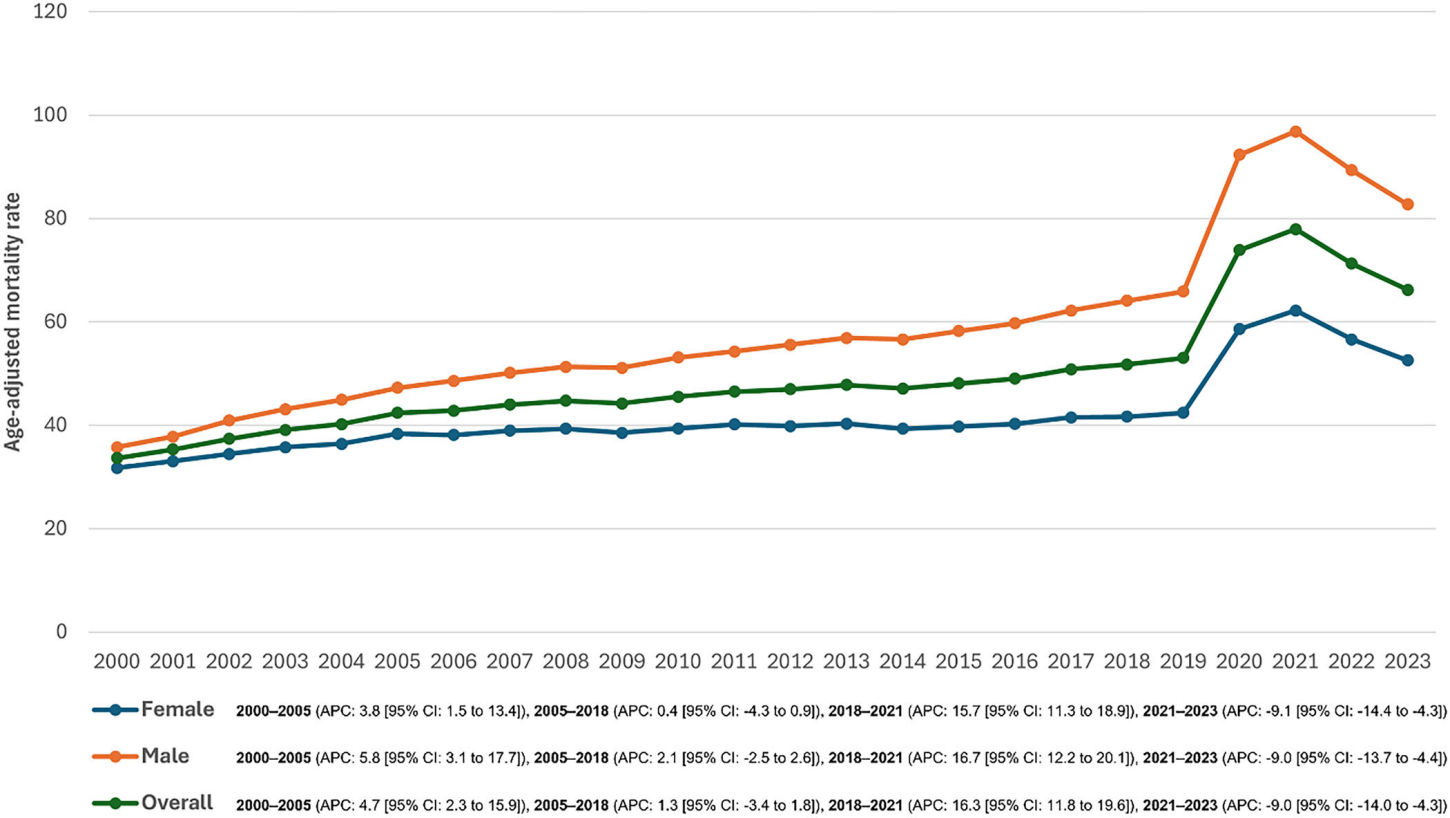
Overall and sex‐stratified diabetes mellitus and hypertension‐related age‐adjusted mortality rates (AAMRs) per 100,000 individuals in the United States, 2000–2023.

The AAMR for hypertension‐related deaths in adults over 25 with DM, was 33.7 in 2000 and rose to 66.2 in 2023. The overall AAMR experienced a moderate increase from 2000 to 2005 (APC: 4.7% [95% CI, 2.3% to 15.9%]), followed by a period of nonsignificant increase from 2005 to 2018 (APC: 1.3% [95% CI, −3.4% to 1.8%]). A sharp increase was observed from 2018 to 2021 (APC: 16.3% [95% CI, 11.8% to 19.6%]). However, from 2021 to 2023, the AAMR declined (APC: −9.0% [95% CI, −14.0% to −4.3%]) (Figure 1, Table S4). A similar trend was seen on sensitivity analysis for hypertension mortality among patients with DM, where the underlying cause of death was restricted to hypertension (Figure S1).

### 3.2 Hypertension‐Related Mortality in Patients With DM, Stratified by Demographics

#### 3.2.1 Gender‐Wise Analysis

From 2000 to 2005, both men and women experienced an increase in AAMR, with a higher rise observed in men (APC: 5.8% [95% CI, 3.1% to 17.7%]) compared to women (APC: 3.8% [95% CI, 1.5% to 13.4%]. Between 2005 and 2018, the AAMR in men showed a minimal change (APC: 2.1% [95% CI, −2.5% to 2.6%]), while in women, it remained largely unchanged (APC: 0.5% [95% CI, −4.3% to 0.9%]).

A sharp increase followed from 2018 to 2021, with AAMR rising significantly in both men (APC: 16.7% [95% CI, 12.2% to 20.1%]) and women (APC: 15.7% [95% CI, 11.3% to 18.9%]). However, from 2021 to 2023, AAMR declined in both sexes, with men experiencing an APC of −9.0% [95% CI, −13.7% to −4.4%] and women an APC of −9.1% [95% CI, −14.4% to −4.3%].

Throughout the study period from 2000 to 2023, the AAMR for men consistently exceeded that of women, with the difference becoming more pronounced over time (Figure 1, Table S4).

#### 3.2.2 Race‐Wise Analysis

**Figure 2:**
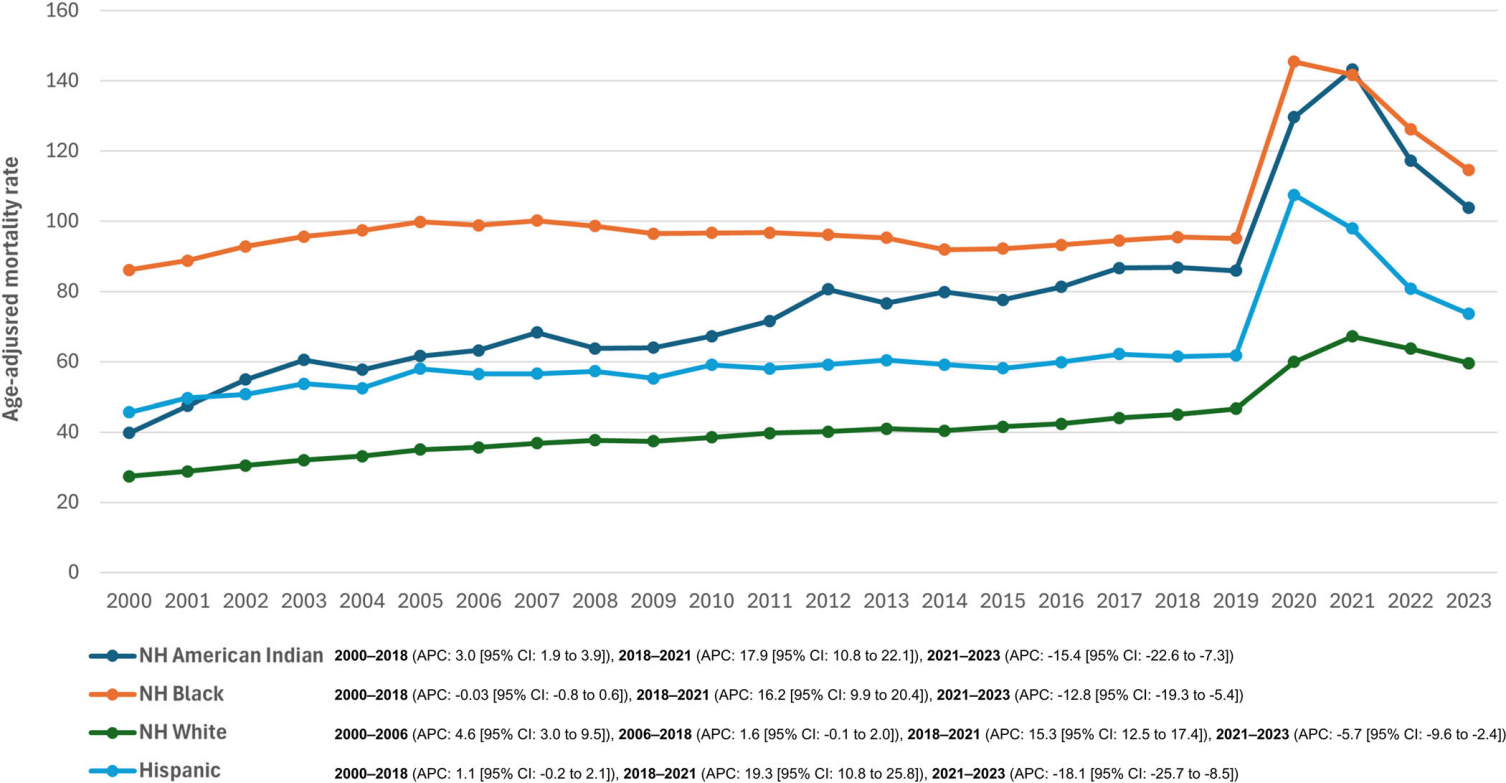
Diabetes mellitus and hypertension‐related age‐adjusted mortality rates (AAMRs) per 100,000 individuals stratified by race in the United States, 2000–2023.

Regarding race/ethnicity, the highest AAMRs were observed among NH Black or African American individuals, followed by NH American Indian or Alaska Native, Hispanic or Latino and NH White individuals (Figure 2, Table S7).

Among NH Black or African American individuals, the AAMR remained constant from 2000 to 2018 (APC: −0.03% [95% CI, −0.8% to 0.6%]), before experiencing a marked increase from 2018 to 2021 (APC: 16.2% [95% CI, 9.9% to 20.4%]). This was followed by a significant decline from 2021 to 2023 (APC: −12.8% [95% CI, −19.3% to −5.4%]), with AAMR values changing from 141.7 to 114.6.

For NH American Indian or Alaska Native individuals, the AAMR increased from 39.8 in 2000 to 86.8 in 2018 (APC: 3.0% [95% CI, 1.9% to 3.9%]), and accelerated significantly from 2018 to 2021 (APC: 17.9% [95% CI, 10.8% to 22.1%]). However, a sharp decline followed from 2021 to 2023 (APC: −15.4% [95% CI, −22.6% to −7.3%]).

Among Hispanic or Latino individuals, the AAMR increased gradually from 2000 to 2018 (APC: 1.1% [95% CI, −0.2% to 2.1%]), followed by a sharp increase from 2018 to 2021 (APC: 19.3% [95% CI, 10.8% to 25.8%]). This was succeeded by a marked decline from 2021 to 2023 (APC: −18.1% [95% CI, −25.7% to −8.5%]).

The AAMR for White individuals increased steadily from 2000 to 2006 (APC: 4.6% [95% CI, 3.0% to 9.5%]) and at a slower rate from 2006 to 2018 (APC: 1.6% [95% CI, −0.1% to 2.0%]). A significant rise occurred from 2018 to 2021 (APC: 15.3% [95% CI, 12.5% to 17.4%]), before declining from 2021 to 2023 (APC: −5.7% [95% CI, −9.6% to −2.4%]).

#### 3.2.3 Age Group‐Wise Analysis

Among younger adults (25–44 years old), the AAMR increased from 1.3 in 2000 to 2.2 in 2008 (APC:6.5% [95% CI, 4.7% to 15.1%]), followed by a period of relative stability, remaining around 2.9 until 2018 (APC: 2.3% [95%CI, −5.2% to 3.4%]). This was followed by a notable rise to 5.0 in 2021 (APC: 20.7% [95%CI, 13.9% to 25.4%]), before declining considerably to 3.6 in 2023 (APC: −16.1% [95% CI, −23.0% to −9.8%]).

In middle‐aged adults (45–64 years old), the AAMR showed a steady increase from 18.6 in 2000 to 30.5 in 2018 (APC: 2.4% [95%CI, 1.8% to 2.9%]). This was followed by a pronounced rise to 47.9 in 2021 (APC: 16.7% [95% CI, 11.7% to 19.7%]), before declining to 37.7 in 2023 (APC: −12.3% [95% CI, −18.1% to −6.9%]).

Among older adults (65+ years old), the AAMR increased from 136.5 in 2000 to 172.1 in 2005 (APC: 4.7% [95% CI, 2.2% to 13.5%]), and continued to rise more gradually, reaching 204.5 in 2018 (APC: 1.1% [95% CI, −2.8% to 1.6%]). This was followed by a substantial increase to 302.6 in 2021 (APC: 15.9% [95% CI, 11.5% to 19.1%]), before decreasing to 264.2 in 2023 (APC: −8.0% [95% CI, −12.6% to −3.5%]) **(**Figure S2, Table S5, Table S6 **)**.

### 3.3 Hypertension‐Related Mortality in Patients With DM, Stratified by Region

#### 3.3.1 Urbanization‐Wise Analysis

Both metropolitan and nonmetropolitan areas saw a continuous increase, but nonmetropolitan areas surpassed metropolitan ones after 2001, maintaining higher AAMRs.

In metropolitan areas, AAMRs rose from 2000 to 2005 (APC: 4.6% [95% CI, 1.9% to 19.6%]), followed by a nonsignificant increase from 2005 to 2018 (APC: 1.1% [95% CI, −5.6% to 1.7%]), before surging from 2018 to 2020 (APC: 18.9% [95% CI, 7.7% to 25.6%]).

Similarly, nonmetropolitan areas experienced a steady increase from 2000 to 2005 (APC: 5.7% [95% CI, 3.5% to 13.5%]), surpassing metropolitan rates after 2001. This was followed by a consistent rise from 2005 to 2018 (APC: 2.1% [95% CI, 0.4% to 2.6%]), and a sharp incline from 2018 to 2020 (APC: 18.8% [95% CI, 12.7% to 22.4%]) (Figure S3, Table S8).

#### 3.3.2 State and Census Region

**Figure 3:**
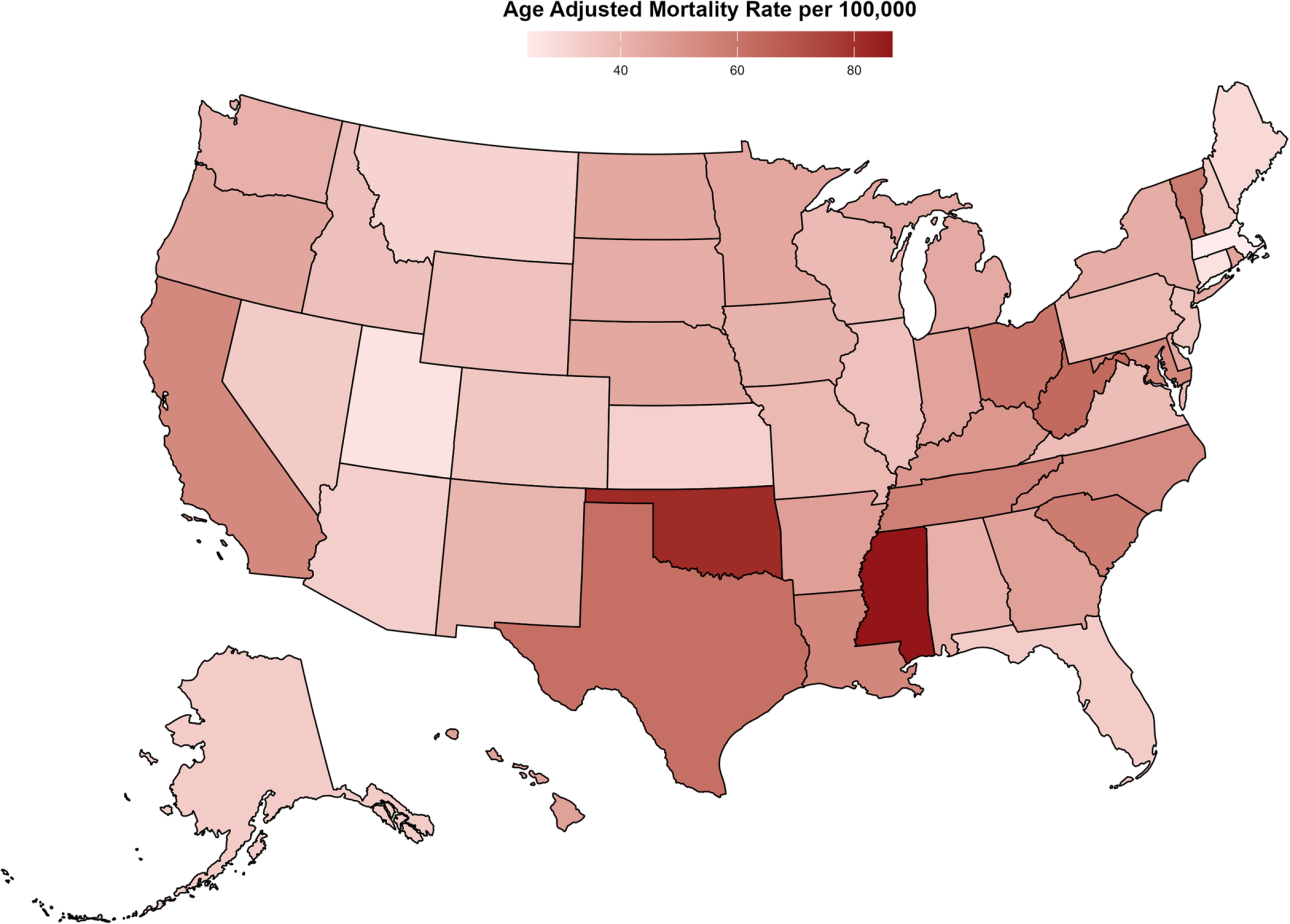
Diabetes mellitus and hypertension‐related age‐adjusted mortality rates (AAMRs) per 100,000 individuals stratified by state in the United States, 2000–2023.

A notable disparity in AAMRs was observed among states, ranging from 24.1 in Massachusetts to 86.5 in Mississippi. States in the top 90th percentile for mortality included Mississippi, District of Columbia, Oklahoma, West Virginia, and Texas, with AAMRs nearly three times higher than those in the lowest 10th percentile, which comprised Massachusetts, Utah, Connecticut, Maine, and Montana (Figure 3, Table S9).

**Figure 4:**
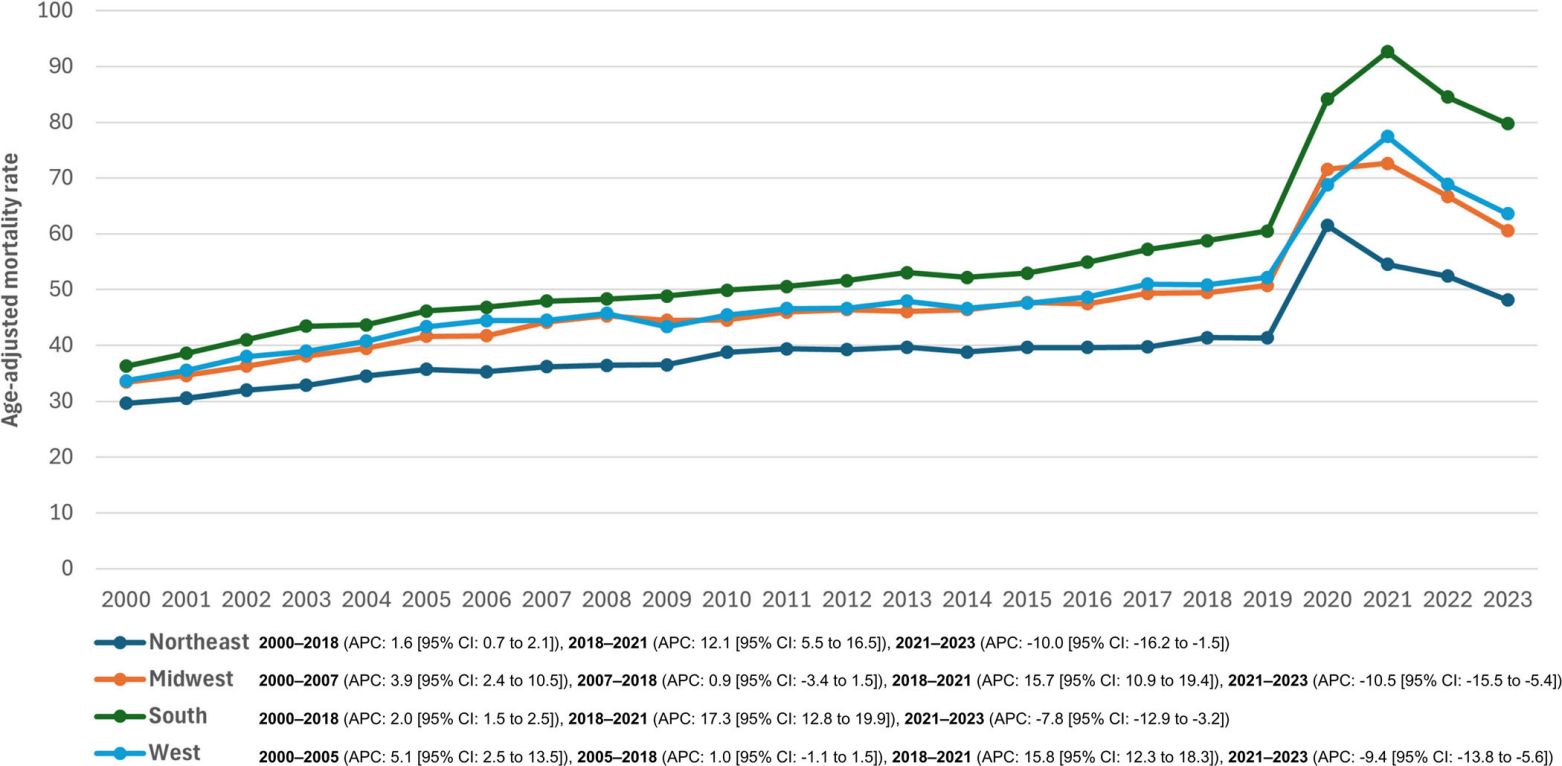
Diabetes mellitus and hypertension‐related age‐adjusted mortality rates (AAMRs) per 100,000 individuals stratified by census region in the United States, 2000–2023.

Over the full study period (2000–2023), significant increases in AAMRs were observed across all Census regions. The South experienced the highest average annual percent change (AAPC: 3.0% [95% CI, 2.6% to 3.4%]), followed by the West (AAPC: 2.8% [95% CI, 2.3% to 3.4%]), the Midwest (AAPC: 2.6% [95% CI, 2.1% to 3.2%]), and the Northeast (AAPC: 1.8% [95% CI, 1.3% to 2.5%]) (Figure 4, Table S10).

## 4 Discussion

This study comprehensively analyzes mortality trends in individuals with DM and hypertension using CDC WONDER data from 2000 to 2023, revealing several key findings. The AAMRs steadily increased up until 2018, followed by a sharp and significant rise through 2021, before declining through 2023. However, despite this recent decrease, the AAMR in 2023 remained significantly higher than in 2000. Mortality trends remained consistent across demographic subgroups, with males showing higher AAMRs than females, with the largest increases observed in patients aged 65 years and older. Among racial groups, NH Black individuals had the highest AAMRs, while NH White individuals had the lowest. Geographically, nonmetropolitan areas experienced higher mortality rates than metropolitan areas, with the South recording the highest AAMRs. States in the 90th percentile exhibited almost three times the AAMR compared to those in the 10th percentile. These findings emphasize the complex interplay between DM and hypertension‐related mortality, highlighting key areas for targeted interventions.

Over the past 24 years, we observed a nearly two‐fold increase in AAMRs for DM and hypertension‐related mortalities. Existing literature highlights that DM exacerbates hypertension‐related mortality by accelerating vascular complications, increasing arterial stiffness, and amplifying the risk of cardiovascular events [21, 22, 23]. The steady increase in mortality rates until 2018 can largely be attributed to the rising prevalence of DM [24], which is driven by multiple interrelated factors. A key contributor is the ongoing obesity epidemic [25], as obesity is a well‐established risk factor for both DM and hypertension [26]. In obesity, visceral adipose tissue plays a crucial role in metabolic dysfunction, as it is strongly linked to insulin resistance, dyslipidemia, hypertension, and chronic systemic inflammation [27, 28, 29]. Similarly, epicardial adipose tissue, which increases with BMI, is associated with elevated fasting insulin, higher diastolic blood pressure, and coronary artery disease [30, 31]. Additionally, poor dietary patterns characterized by excessive consumption of ultra‐processed foods and sugar‐sweetened beverages [32], combined with increasingly sedentary lifestyles, have further contributed to the rising prevalence of these comorbidities [33].

The steep rise in AAMRs during 2020–2021 can largely be attributed to the impact of the COVID‐19 pandemic, a conclusion further supported by the subsequent decline in AAMRs through 2023 as the pandemic subsided. Li et al. reported that individuals with DM and hypertension were among the most vulnerable populations, as both conditions were major risk factors for severe COVID‐19 outcomes, including mortality [34]. Laboratory and clinical studies indicate that the severity and mortality of COVID‐19 in patients with DM and hypertension may be driven by ACE2 overexpression in adipose tissue, impaired immune function, heightened pro‐inflammatory response, and cytokine storm [35, 36]. Beyond direct physiological impacts, the pandemic also disrupted routine healthcare services, delaying medical checkups, screenings, and chronic disease management [37]. Many individuals faced limited access to medications and overwhelmed hospital systems, leading to worsened disease progression and complications [38]. Additionally, socioeconomic and racial disparities were magnified during the pandemic, with older adults, racial minorities, and rural residents experiencing the most pronounced increases in mortality [39]. The recent decline in AAMRs through 2023 may reflect advancements in medical treatments, improved public health initiatives, and greater awareness of cardiovascular risk management. Additionally, this decline could be due in part to the pandemic becoming less severe and its disruptions to healthcare systems easing, allowing for better disease management and access to routine medical care.

Although our findings cannot confer causation, several previous analyses have shown a pathophysiological association between DM and hypertension and vice versa. Both conditions share mechanisms that contribute to cardiovascular and renal complications, with insulin resistance and renin‐angiotensin‐aldosterone system (RAAS) dysregulation playing central roles. Hyperinsulinemia increases renal sodium reabsorption, causing fluid retention and elevated blood pressure [40], while hyperglycemia‐induced hyperosmolarity expands circulatory volume, further promoting hypertension [41]. Hyperinsulinemia also activates the sympathetic nervous system, increasing renin release, cardiac output, and vascular resistance [42, 43]. Chronic hyperglycemia and hyperinsulinemia lead to vascular remodeling and arterial stiffness, sustaining hypertension [44]. Conversely, hypertension accelerates diabetic nephropathy by increasing glomerular pressure and proteinuria, worsening renal function [45]. Inflammation and oxidative stress, common to both diseases, impair endothelial function, worsen insulin resistance, and disrupt vascular homeostasis [46, 47]. Endothelial dysfunction further reduces nitric oxide bioavailability, limiting vasodilation and increasing vascular stiffness [48, 49]. This bidirectional relationship significantly heightens the risk of cardiovascular events, kidney failure, and mortality.

We also report gender disparity in AAMRs, with men experiencing higher mortality rates than women. Our findings align with existing literature, which consistently shows that men have a higher prevalence of both DM and hypertension [50, 51]. This disparity stems from both biological and behavioral factors. Sex hormones play a key role, as estrogen provides protective cardiovascular effects in premenopausal women, improving endothelial function and reducing blood pressure [52, 53]. In contrast, testosterone has been associated with enhanced sodium retention, contributing to a higher risk of hypertension in men [54]. Men also accumulate more visceral fat, which is strongly linked to insulin resistance, dyslipidemia, and hypertension [55]. Additionally, studies indicate that men with a family history of DM are more prone to insulin resistance than women [56]. Behavioral factors further contribute to this disparity, as men are more likely to smoke, consume excessive alcohol, and engage in other lifestyle behaviors that worsen DM and hypertension [57]. Moreover, men are less likely to seek preventive healthcare, leading to delayed diagnosis and inadequate disease management [58].

Regarding race, NH Black individuals had the highest mortality rates among ethnic groups, followed by American Indians, Hispanics/Latinos, and NH Whites, who had the lowest AAMRs. However, all racial groups experienced an increase in AAMRs over the study period. This disparity in DM‐hypertension‐related mortality can be attributed to several factors. Black individuals have a higher genetic predisposition to hypertension, characterized by increased salt sensitivity, leading to earlier‐onset and more severe hypertension [59]. Similarly, insulin resistance is more prevalent in Black and Hispanic populations, increasing their risk of developing type 2 diabetes [60, 61]. Socioeconomic disparities also play a crucial role, as Black, Hispanic, and American Indian populations are more likely to face barriers to healthcare access, leading to delayed diagnosis and inadequate management of DM and hypertension [62]. Limited access to preventive care, financial constraints, and under‐resourced healthcare systems contribute to poorer health outcomes. Furthermore, chronic psychological stress from racial discrimination, financial instability, and neighborhood disadvantage exacerbates disease progression by elevating cortisol levels and activating the sympathetic nervous system, further worsening blood pressure control and metabolic health [63, 64].

All age groups experienced an increase in AAMRs over time, with older adults experiencing significantly higher mortality rates than younger and middle‐aged adults. While the rise was more pronounced in older adults, younger adults maintained consistently lower AAMRs. However, over the study period, AAMRs in younger adults also increased, reflecting a concerning trend driven by rising obesity rates, poor dietary habits, increased smoking and alcohol consumption, and increasingly sedentary lifestyles [65]. Additionally, higher stress levels, inadequate sleep, and a growing prevalence of metabolic syndrome among younger adults contribute to the early onset of hypertension and type 2 diabetes, accelerating disease progression and increasing mortality risk [66, 67]. In older adults, aging‐related vascular changes, such as vascular stiffening, endothelial dysfunction, and reduced baroreceptor sensitivity, further worsen hypertension and impaired glucose metabolism [68]. Moreover, prolonged exposure to metabolic risk factors leads to more advanced stages of DM and hypertension, increasing susceptibility to cardiovascular disease, kidney failure, and stroke [69]. Furthermore, immune system decline and chronic inflammation exacerbate DM‐hypertension‐related complications [70], leading to greater vulnerability to infections, including COVID‐19, which disproportionately affected this age group. The pandemic further amplified mortality rates among older adults due to higher rates of hospitalization, severe complications, and healthcare disruptions.

Geographic disparities were evident, with significant regional variations in AAMRs. The highest mortality rates were reported in the South, followed by the West, Midwest, and Northeast, with Mississippi and the District of Columbia exhibiting particularly high AAMRs. Additionally, mortality rates were substantially higher in nonmetropolitan areas compared to metropolitan regions, reflecting a long‐standing healthcare divide. Communities in nonmetropolitan areas often face healthcare provider shortages, fewer specialized services, and longer travel distances to medical facilities, leading to delayed diagnoses and inadequate disease management [71]. The burden is further compounded by higher rates of obesity, smoking, and physical inactivity, all of which contribute to worsening DM and hypertension outcomes [72]. Socioeconomic challenges, including higher poverty rates, lower insurance coverage, and limited access to preventive care, place additional strain on these communities [73]. These vulnerabilities were magnified during the COVID‐19 pandemic, as nonmetropolitan populations were disproportionately affected, leading to higher mortality rates.

Addressing the rising burden of DM‐hypertension mortality requires targeted interventions that prioritize high‐risk populations and improve access to early diagnosis, prevention, and treatment. Our findings highlight persistent racial, geographic, and gender disparities, underscoring the urgent need for equitable healthcare solutions in underserved communities. Guidelines from the American Diabetes Association (ADA) and American Heart Association (AHA) recommend a multifaceted approach, including lifestyle changes—such as diet, physical activity, and weight management—and pharmacologic therapy like renin‐angiotensin system inhibitors, thiazide diuretics, and calcium channel blockers for blood pressure control in diabetic patients with hypertension [74, 75]. Yet many individuals, especially in nonmetropolitan areas and racial minority groups, face challenges accessing timely care due to financial barriers, provider shortages, and limited healthcare infrastructure. Expanding insurance coverage, improving medication affordability, and increasing healthcare access are vital to reducing these disparities. Newer antihyperglycemic agents, such as GLP‐1 agonists and SGLT2 inhibitors, offer promise by lowering blood pressure while improving glucose control [76]. However, further research—particularly randomized, multi‐center clinical trials—is needed to better define their role in managing DM‐hypertension comorbidity. A coordinated effort involving clinical care, public health, and policy reforms is essential to bridge these gaps and improve health outcomes.

### 4.1 Limitations

Our study has several limitations. The data relied solely on ICD‐10 codes, which may be subject to misclassification or omissions, potentially impacting the accuracy of mortality trends. Key variables, such as socioeconomic status, were not available, despite being a crucial determinant of healthcare access and health outcomes. The database also lacks clinical and laboratory data, as well as treatment history, which could have provided a more comprehensive understanding of disease progression and management. Additionally, urbanization data was unavailable after 2020, limiting our ability to assess recent trends in metropolitan and nonmetropolitan disparities.

## 5 Conclusion

Our study reveals a steady rise in DM‐hypertension mortality, with a sharp increase during the COVID‐19 pandemic. Men, NH Black individuals, older adults, and nonmetropolitan populations faced the highest mortality burden, with the South reporting the highest AAMRs. These findings highlight the impact of socioeconomic and healthcare disparities on disease outcomes. Addressing these gaps through targeted interventions, improved healthcare access, and fair, widespread prevention efforts is crucial to reducing mortality and improving long‐term health outcomes.

## Ethics Statement

The authors have nothing to report.

## Conflicts of Interest

The authors declare no conflicts of interest.

## Supporting information

Supplementary Material.

## Data Availability

The data that support the findings of this study are openly available in CDC‐WONDER at https://wonder.cdc.gov/. The data supporting the findings of this study were obtained from the CDC WONDER online database (Centers for Disease Control and Prevention Wide‐ranging Online Data for Epidemiologic Research). The datasets used and analyzed during the current study are publicly available and can be accessed at [CDC WONDER] (https://wonder.cdc.gov).
