# Supplementary material for: A Rising Crisis: Escalating Burden of Diabetes Mellitus and Hypertension‐Related Mortality Trends in the United States, 2000–2023
Source: Clin Cardiol. 2025 Jul 2;48(7):e70167. doi: 10.1002/clc.70167 (PMC12217654; doi:10.1002/clc.70167)
Supplement: Supplementary file 1 — Supplementary Material. [file CLC-48-e70167-s001.docx]

**Supplementary Material**

| **Year** | **Overall** | **Male** | **Female** | **NH Black  or African American** | **NH White** | **Hispanic or Latino** | **Population** |
| --- | --- | --- | --- | --- | --- | --- | --- |
| **2000** | 60214 | 26315 | 33899 | 13098 | 41069 | 4140 | 181984640 |
| **2001** | 64252 | 28427 | 35825 | 13791 | 43579 | 4767 | 184305128 |
| **2002** | 69116 | 31346 | 37770 | 14724 | 46744 | 5175 | 186208028 |
| **2003** | 73507 | 33812 | 39695 | 15494 | 49664 | 5799 | 188090429 |
| **2004** | 76830 | 35869 | 40961 | 16085 | 51997 | 5947 | 190205384 |
| **2005** | 82564 | 38635 | 43929 | 16943 | 55709 | 6913 | 192551384 |
| **2006** | 85087 | 40700 | 44387 | 17223 | 57545 | 7113 | 195019359 |
| **2007** | 89108 | 42936 | 46172 | 17913 | 60379 | 7465 | 197403777 |
| **2008** | 92586 | 45017 | 47569 | 18115 | 62903 | 7953 | 199795090 |
| **2009** | 93394 | 46045 | 47349 | 18224 | 63263 | 8111 | 202107016 |
| **2010** | 97757 | 48630 | 49127 | 18751 | 66009 | 8965 | 203891983 |
| **2011** | 102687 | 51369 | 51318 | 19436 | 69443 | 9434 | 206592936 |
| **2012** | 106235 | 54128 | 52107 | 19941 | 71430 | 10133 | 208826037 |
| **2013** | 110602 | 56970 | 53632 | 20459 | 74187 | 10983 | 211085314 |
| **2014** | 111798 | 58463 | 53335 | 20467 | 74472 | 11463 | 213809280 |
| **2015** | 116666 | 61626 | 55040 | 21222 | 77595 | 11974 | 216553817 |
| **2016** | 121531 | 64722 | 56809 | 22158 | 80462 | 12855 | 218641417 |
| **2017** | 129080 | 69160 | 59920 | 23214 | 85079 | 14109 | 221447331 |
| **2018** | 134212 | 72988 | 61224 | 24064 | 88461 | 14519 | 223311190 |
| **2019** | 140358 | 76787 | 63571 | 24647 | 93125 | 15220 | 224981167 |
| **2020** | 199038 | 109991 | 89047 | 38798 | 121048 | 27875 | 226635013 |
| **2021** | 206069 | 114667 | 91402 | 37236 | 130564 | 26121 | 228238412 |
| **2022** | 196198 | 108497 | 87701 | 33805 | 129009 | 22091 | 229508599 |
| **2023** | 183779 | 102224 | 81555 | 31283 | 120835 | 20840 | 231529762 |
| **Total** | 2742668 | 1419324 | 1323344 | 517091 | 1814571 | 279965 | 4982722493 |

**Table S1**. Overall and sex-stratified diabetes mellitus and hypertension-related deaths per 100,000 in the United States from 2000 to 2023.

| **Year** | **Medical Facility - Inpatient** | **Medical Facility - Outpatient or ER** | **Medical Facility - Dead on Arrival** | **Decedent’s Home** | **Hospice Facility** | **Nursing Home/Long-Term Care Facility** | **Other** | **Place of Death Unknown** |
| --- | --- | --- | --- | --- | --- | --- | --- | --- |
| **2000** | 22783 | 7467 | 1057 | 14148 | Missing | 12887 | 1586 | 12 |
| **2001** | 24010 | 7605 | 1181 | 15142 | Missing | 14126 | 1887 | 18 |
| **2002** | 24853 | 8220 | 1113 | 17017 | Missing | 15397 | 2194 | 14 |
| **2003** | 25900 | 8760 | 1056 | 18629 | 71 | 16335 | 2539 | 217 |
| **2004** | 26105 | 8983 | 1001 | 20223 | 160 | 17271 | 2865 | 222 |
| **2005** | 27544 | 9622 | 943 | 22231 | 588 | 18542 | 2886 | 208 |
| **2006** | 27373 | 9858 | 949 | 23785 | 1018 | 18873 | 2984 | 247 |
| **2007** | 28244 | 9878 | 953 | 25209 | 1605 | 19751 | 3271 | 197 |
| **2008** | 28184 | 10036 | 842 | 26775 | 2169 | 19837 | 3196 | 1547 |
| **2009** | 26287 | 10831 | 806 | 27761 | 2269 | 19449 | 3529 | 2462 |
| **2010** | 27311 | 11745 | 997 | 30587 | 2665 | 20453 | 3935 | 64 |
| **2011** | 27879 | 12046 | 914 | 32695 | 3273 | 21587 | 4234 | 59 |
| **2012** | 27201 | 12770 | 868 | 34602 | 3938 | 22148 | 4647 | 61 |
| **2013** | 27415 | 12988 | 935 | 37731 | 4057 | 22476 | 4935 | 65 |
| **2014** | 26949 | 13234 | 785 | 39192 | 4605 | 22464 | 4490 | 79 |
| **2015** | 27794 | 13656 | 808 | 41469 | 5196 | 23088 | 4623 | 32 |
| **2016** | 28525 | 13708 | 822 | 44248 | 5865 | 23450 | 4889 | 24 |
| **2017** | 29199 | 14465 | 745 | 47654 | 6513 | 25068 | 5408 | 28 |
| **2018** | 29602 | 14840 | 679 | 50854 | 6632 | 25901 | 5686 | 18 |
| **2019** | 30387 | 15119 | 709 | 54370 | 7421 | 26058 | 6267 | 27 |
| **2020** | 53772 | 18925 | 802 | 76541 | 8396 | 32052 | 8517 | 33 |

**Table S2.** Diabetes mellitus and hypertension-related mortality per 100,000 stratified by place of death and year in the United States from 2000 to 2020.

| **Place of Death** | **Deaths** | **% of Total Deaths** |
| --- | --- | --- |
| **Medical Facility** | 875,075 | 40.09% |
| **Decedent's Home** | 707,593 | 32.41% |
| **Hospice Facility** | 66,441 | 3.04% |
| **Nursing home/long-term care** | 442,957 | 20.29% |
| **Other** | 85,309 | 3.91% |
| **Place of Death Unknown** | 5,643 | 0.26% |
| **Total** | 2,183,018 | 100% |

**Table S3.** Diabetes mellitus and hypertension-related mortality per 100,000 stratified by place of death in the United States from 2000 to 2020.

| **Age-Adjusted Rate (95% CI)** | | | |
| --- | --- | --- | --- |
| **Year** | **Female** | **Male** | **Overall** |
| **2000** | 31.78 (31.44 – 32.12) | 35.78 (35.34 – 36.22) | 33.67 (33.40 – 33.94) |
| **2001** | 33.05 (32.71 – 33.40) | 37.82 (37.37 – 38.26) | 35.35 (35.07 – 35.62) |
| **2002** | 34.45 (34.10 – 34.79) | 40.94 (40.48 – 41.40) | 37.40 (37.13 – 37.68) |
| **2003** | 35.74 (35.39 – 36.10) | 43.12 (42.65 – 43.59) | 39.11 (38.83 – 39.39) |
| **2004** | 36.41 (36.05 – 36.76) | 44.92 (44.45 – 45.39) | 40.24 (39.96 – 40.53) |
| **2005** | 38.38 (38.02 – 38.75) | 47.25 (46.77 – 47.73) | 42.41 (42.12 – 42.70) |
| **2006** | 38.15 (37.79 – 38.51) | 48.60 (48.12 – 49.08) | 42.83 (42.54 – 43.12) |
| **2007** | 38.97 (38.61 – 39.32) | 50.13 (49.64 – 50.61) | 44.02 (43.73 – 44.31) |
| **2008** | 39.37 (39.01 – 39.73) | 51.30 (50.82 – 51.78) | 44.74 (44.45 – 45.03) |
| **2009** | 38.58 (38.23 – 38.93) | 51.14 (50.67 – 51.62) | 44.25 (43.96 – 44.53) |
| **2010** | 39.41 (39.06 – 39.76) | 53.13 (52.65 – 53.62) | 45.52 (45.24 – 45.81) |
| **2011** | 40.19 (39.84 – 40.55) | 54.30 (53.82 – 54.78) | 46.51 (46.23 – 46.80) |
| **2012** | 39.88 (39.53 – 40.23) | 55.62 (55.14 – 56.10) | 46.99 (46.71 – 47.28) |
| **2013** | 40.33 (39.98 – 40.68) | 56.91 (56.43 – 57.38) | 47.81 (47.52 – 48.09) |
| **2014** | 39.34 (39.00 – 39.68) | 56.63 (56.16 – 57.10) | 47.14 (46.86 – 47.42) |
| **2015** | 39.78 (39.44 – 40.11) | 58.27 (57.80 – 58.74) | 48.12 (47.84 – 48.40) |
| **2016** | 40.29 (39.95 – 40.63) | 59.74 (59.27 – 60.21) | 49.04 (48.76 – 49.32) |
| **2017** | 41.53 (41.19 – 41.87) | 62.22 (61.74 – 62.69) | 50.85 (50.57 – 51.13) |
| **2018** | 41.66 (41.33 – 42.00) | 64.13 (63.66 – 64.61) | 51.79 (51.51 – 52.07) |
| **2019** | 42.41 (42.08 – 42.75) | 65.90 (65.43 – 66.38) | 53.02 (52.74 – 53.30) |
| **2020** | 58.64 (58.25 – 59.04) | 92.34 (91.78 – 92.89) | 73.88 (73.55 – 74.20) |
| **2021** | 62.23 (61.82 – 62.64) | 96.86 (96.29 – 97.44) | 77.93 (77.59 – 78.27) |
| **2022** | 56.64 (56.26 – 57.02) | 89.35 (88.80 – 89.89) | 71.30 (70.98 – 71.62) |
| **2023** | 52.59 (52.23 – 52.96) | 82.75 (82.23 – 83.27) | 66.23 (65.92 – 66.54) |
| **Total** | 41.58 (41.23 – 41.94) | 58.73 (58.23 – 59.24) | 50.71 (50.40 – 51.03) |

**Table S4.** Overall and sex-stratified diabetes mellitus and hypertension-related AAMR per 100,000 in the United States from 2000 to 2023.

| **Crude Rate per 100,000 (95% CI)** | | | | |
| --- | --- | --- | --- | --- |
| **Year** | **25-44** | | **45-64** | **65-85+** |
| **2000** | 1.31 (1.23 - 1.39) | 18.53 (18.19 - 18.87) | | 136.09 (134.87 - 137.31) |
| **2001** | 1.40 (1.32 - 1.48) | 19.12 (18.78 - 19.46) | | 143.78 (142.53 - 145.03) |
| **2002** | 1.54 (1.46 - 1.63) | 20.46 (20.12 - 20.80) | | 152.51 (151.23 - 153.79) |
| **2003** | 1.77 (1.68 - 1.86) | 21.75 (21.40 - 22.10) | | 159.11 (157.80 - 160.41) |
| **2004** | 1.73 (1.64 - 1.82) | 22.33 (21.99 - 22.68) | | 164.49 (163.17 - 165.81) |
| **2005** | 1.93 (1.83 - 2.02) | 23.58 (23.23 - 23.93) | | 173.88 (172.53 - 175.23) |
| **2006** | 1.93 (1.83 - 2.02) | 24.44 (24.09 - 24.80) | | 175.20 (173.85 - 176.54) |
| **2007** | 2.07 (1.97 - 2.16) | 25.06 (24.71 - 25.42) | | 180.01 (178.66 - 181.36) |
| **2008** | 2.12 (2.02 - 2.22) | 25.89 (25.53 - 26.24) | | 181.77 (180.43 - 183.11) |
| **2009** | 2.24 (2.14 - 2.34) | 25.84 (25.49 - 26.19) | | 178.71 (177.39 - 180.02) |
| **2010** | 2.22 (2.12 - 2.32) | 26.81 (26.45 - 27.16) | | 183.99 (182.66 - 185.31) |
| **2011** | 2.23 (2.12 - 2.33) | 28.13 (27.77 - 28.49) | | 187.39 (186.07 - 188.71) |
| **2012** | 2.38 (2.27 - 2.48) | 28.40 (28.03 - 28.76) | | 187.13 (185.84 - 188.42) |
| **2013** | 2.37 (2.27 - 2.47) | 29.66 (29.29 - 30.03) | | 187.88 (186.61 - 189.15) |
| **2014** | 2.54 (2.43 - 2.65) | 30.14 (29.77 - 30.51) | | 182.69 (181.46 - 183.92) |
| **2015** | 2.56 (2.45 - 2.67) | 30.87 (30.49 - 31.24) | | 185.39 (184.17 - 186.62) |
| **2016** | 2.56 (2.45 - 2.67) | 31.94 (31.56 - 32.33) | | 187.71 (186.50 - 188.92) |
| **2017** | 2.52 (2.41 - 2.62) | 33.30 (32.91 - 33.69) | | 194.29 (193.07 - 195.50) |
| **2018** | 2.64 (2.53 - 2.75) | 33.89 (33.49 - 34.28) | | 197.37 (196.16 - 198.57) |
| **2019** | 2.70 (2.59 - 2.81) | 34.98 (34.57 - 35.38) | | 201.36 (200.17 - 202.56) |
| **2020** | 4.10 (3.97 - 4.23) | 49.93 (49.45 - 50.41) | | 276.85 (275.47 - 278.24) |
| **2021** | 4.66 (4.52 - 4.81) | 53.59 (53.10 - 54.09) | | 281.44 (280.04 - 282.83) |
| **2022** | 3.86 (3.73 - 3.99) | 46.85 (46.38 - 47.31) | | 266.63 (265.30 - 267.96) |
| **2023** | 3.40 (3.28 - 3.52) | 42.21 (41.77 - 42.65) | | 246.35 (245.09 - 247.61) |

**Table S5.** Diabetes mellitus and hypertension-related AAMR per 100,000 stratified by age group in the United States from 2000 to 2023.

| **Age Group** | **Interval of Time** | **APC (95% CI)** |
| --- | --- | --- |
| **25-44** | 2000 to 2008 | 6.51 (4.71 to 15.13 |
|  | 2008 to 2018 | 2.28 (-5.19 to 3.36) |
|  | 2018 to 2021 | 20.66 (13.93 to 25.36) |
|  | 2021 to 2023 | -16.07 (-23.02 to -9.82) |
| **45-64** | 2000 to 2018 | 2.41 (1.81 to 2.92) |
|  | 2018 to 2021 | 16.72 (11.68 to 19.65) |
|  | 2021 to 2023 | -12.24 (-18.06 to -6.90) |
| **65-85+** | 2000 to 2005 | 4.67 (2.16 to 13.51) |
|  | 2005 to 2018 | 1.11 (-2.84 to 1.59) |
|  | 2018 to 2021 | 15.86 (11.51 to 19.12) |
|  | 2021 to 2023 | -7.95 (-12.58 to -3.52) |

**Table S6.** Summary APCs of diabetes mellitus and hypertension-related mortality crude rate per 100,000 stratified by age group in the United States from 2000 to 2023.

| **Year** | **American Indian or Alaska Native** | **NH Black or African American** | **NH White** | **Hispanic or Latino** |
| --- | --- | --- | --- | --- |
| **2000** | 39.81 (35.29 – 44.33) | 86.09 (84.59 – 87.58) | 27.43 (27.17 – 27.70) | 45.65 (44.20 – 47.10) |
| **2001** | 47.45 (42.46 – 52.45) | 88.76 (87.26 – 90.26) | 28.78 (28.51 – 29.05) | 49.62 (48.15 – 51.09) |
| **2002** | 54.90 (49.63 – 60.18) | 92.82 (91.29 – 94.34) | 30.51 (30.23 – 30.78) | 50.66 (49.22 – 52.10) |
| **2003** | 60.46 (54.94 – 65.97) | 95.62 (94.09 – 97.15) | 32.02 (31.74 – 32.30) | 53.70 (52.26 – 55.15) |
| **2004** | 57.67 (52.41 – 62.93) | 97.36 (95.82 – 98.89) | 33.12 (32.83 – 33.40) | 52.41 (51.02 – 53.80) |
| **2005** | 61.59 (56.19 – 66.98) | 99.83 (98.29 – 101.36) | 35.03 (34.74 – 35.32) | 57.92 (56.50 – 59.34) |
| **2006** | 63.20 (57.91 – 68.49) | 98.82 (97.31 – 100.33) | 35.63 (35.34 – 35.92) | 56.50 (55.13 – 57.87) |
| **2007** | 68.28 (62.79 – 73.77) | 100.18 (98.68 – 101.68) | 36.83 (36.54 – 37.13) | 56.55 (55.21 – 57.88) |
| **2008** | 63.72 (58.55 – 68.90) | 98.65 (97.18 – 100.13) | 37.71 (37.41 – 38.01) | 57.22 (55.91 – 58.53) |
| **2009** | 63.99 (58.87 – 69.12) | 96.49 (95.05 – 97.93) | 37.40 (37.10 – 37.69) | 55.23 (53.98 – 56.48) |
| **2010** | 67.28 (62.10 – 72.45) | 96.70 (95.28 – 98.13) | 38.50 (38.21 – 38.80) | 59.08 (57.80 – 60.35) |
| **2011** | 71.57 (66.49 – 76.65) | 96.77 (95.37 – 98.17) | 39.73 (39.43 – 40.03) | 58.01 (56.80 – 59.23) |
| **2012** | 80.59 (75.30 – 85.88) | 96.11 (94.74 – 97.49) | 40.10 (39.80 – 40.39) | 59.17 (57.98 – 60.36) |
| **2013** | 76.62 (71.60 – 81.65) | 95.28 (93.93 – 96.62) | 40.95 (40.65 – 41.25) | 60.39 (59.22 – 61.56) |
| **2014** | 79.85 (74.89 – 84.81) | 91.90 (90.60 – 93.20) | 40.43 (40.14 – 40.73) | 59.15 (58.02 – 60.27) |
| **2015** | 77.61 (72.83 – 82.38) | 92.21 (90.93 – 93.48) | 41.51 (41.22 – 41.81) | 58.07 (56.99 – 59.14) |
| **2016** | 81.29 (76.49 – 86.09) | 93.23 (91.97 – 94.50) | 42.36 (42.07 – 42.66) | 59.81 (58.74 – 60.88) |
| **2017** | 86.65 (81.83 – 91.47) | 94.50 (93.25 – 95.75) | 44.03 (43.72 – 44.33) | 62.12 (61.06 – 63.18) |
| **2018** | 86.83 (82.14 – 91.53) | 95.45 (94.21 – 96.69) | 44.99 (44.69 – 45.30) | 61.44 (60.41 – 62.47) |
| **2019** | 85.92 (81.33 – 90.52) | 95.11 (93.89 – 96.33) | 46.65 (46.34 – 46.95) | 61.77 (60.76 – 62.78) |
| **2020** | 129.65 (124.15 – 135.15) | 145.44 (143.95 – 146.93) | 59.91 (59.56 – 60.25) | 107.48 (106.18 – 108.78) |
| **2021** | 143.25 (137.20 – 149.30) | 141.73 (140.24 – 143.22) | 67.18 (66.80 – 67.55) | 97.96 (96.72 – 99.19) |
| **2022** | 117.30 (111.99 – 122.60) | 126.26 (124.87 – 127.64) | 63.71 (63.36 – 64.07) | 80.72 (79.62 – 81.82) |
| **2023** | 103.82 (98.89 – 108.75) | 114.59 (113.29 – 115.90) | 59.59 (59.24 – 59.93) | 73.58 (72.55 – 74.62) |

**Table S7.** Diabetes mellitus and hypertension-related AAMR per 100,000 stratified by race in the United States from 2000 to 2023.

| **Age-Adjusted Rate (95% CI)** | | |
| --- | --- | --- |
| **Year** | **Metropolitan** | **Non-Metropolitan** |
| **2000** | 33.68 (33.38 – 33.97) | 33.57 (32.95 – 34.19) |
| **2001** | 35.30 (35.00 – 35.61) | 35.50 (34.87 – 36.13) |
| **2002** | 37.14 (36.83 – 37.45) | 38.51 (37.85 – 39.17) |
| **2003** | 38.73 (38.42 – 39.05) | 40.68 (40.01 – 41.35) |
| **2004** | 39.83 (39.52 – 40.15) | 42.01 (41.33 – 42.69) |
| **2005** | 41.90 (41.59 – 42.22) | 44.73 (44.03 – 45.42) |
| **2006** | 42.52 (42.20 – 42.84) | 44.42 (43.73 – 45.11) |
| **2007** | 43.26 (42.94 – 43.58) | 47.33 (46.62 – 48.04) |
| **2008** | 43.94 (43.62 – 44.26) | 48.41 (47.70 – 49.13) |
| **2009** | 43.27 (42.96 – 43.58) | 48.91 (48.20 – 49.63) |
| **2010** | 44.68 (44.37 – 45.00) | 49.66 (48.94 – 50.37) |
| **2011** | 45.61 (45.30 – 45.93) | 51.10 (50.38 – 51.83) |
| **2012** | 45.98 (45.66 – 46.29) | 51.98 (51.25 – 52.70) |
| **2013** | 46.71 (46.40 – 47.02) | 53.23 (52.50 – 53.96) |
| **2014** | 46.12 (45.81 – 46.42) | 52.43 (51.71 – 53.15) |
| **2015** | 46.70 (46.40 – 47.01) | 55.21 (54.48 – 55.95) |
| **2016** | 47.71 (47.41 – 48.02) | 55.89 (55.15 – 56.62) |
| **2017** | 49.19 (48.88 – 49.49) | 59.39 (58.64 – 60.15) |
| **2018** | 49.97 (49.67 – 50.28) | 61.14 (60.38 – 61.90) |
| **2019** | 50.82 (50.52 – 51.13) | 64.60 (63.83 – 65.38) |
| **2020** | 71.70 (71.35 – 72.05) | 85.59 (84.70 – 86.48) |

**Table S8.** Diabetes mellitus and hypertension-related AAMR per 100,000 stratified by Urban-Rural classification in the United States from 2000 to 2020.

| **State** | **Age-Adjusted Rate (95% CI)** |
| --- | --- |
| **Mississippi** | 86.48 (85.61 – 87.36) |
| **District of Columbia** | 86.19 (84.21 – 88.18) |
| **Oklahoma** | 81.67 (80.93 – 82.41) |
| **West Virginia** | 63.53 (62.68 – 64.39) |
| **Texas** | 61.61 (61.34 – 61.89) |
| **Ohio** | 60.22 (59.87 – 60.57) |
| **Vermont** | 58.56 (57.11 – 60.02) |
| **South Carolina** | 57.78 (57.22 – 58.34) |
| **Tennessee** | 56.61 (56.13 – 57.08) |
| **Louisiana** | 54.78 (54.21 – 55.34) |
| **Maryland** | 54.49 (53.99 – 54.98) |
| **California** | 53.78 (53.58 – 53.98) |
| **North Carolina** | 53.43 (53.05 – 53.81) |
| **Kentucky** | 49.43 (48.89 – 49.96) |
| **Arkansas** | 47.06 (46.44 – 47.68) |
| **Georgia** | 46.34 (45.95 – 46.72) |
| **Hawaii** | 46.31 (45.43 – 47.20) |
| **Rhode Island** | 45.90 (44.92 – 46.87) |
| **Indiana** | 45.84 (45.42 – 46.26) |
| **Oregon** | 44.79 (44.26 – 45.31) |
| **Minnesota** | 44.71 (44.25 – 45.16) |
| **North Dakota** | 44.40 (43.20 – 45.60) |
| **Nebraska** | 44.39 (43.63 – 45.15) |
| **Michigan** | 43.85 (43.53 – 44.18) |
| **Delaware** | 43.73 (42.65 – 44.80) |
| **New York** | 42.85 (42.62 – 43.08) |
| **South Dakota** | 42.75 (41.67 – 43.83) |
| **Alabama** | 41.96 (41.50 – 42.43) |
| **Washington** | 41.72 (41.32 – 42.13) |
| **Iowa** | 41.14 (40.60 – 41.68) |
| **New Mexico** | 40.16 (39.45 – 40.87) |
| **Missouri** | 39.39 (39.00 – 39.79) |
| **Pennsylvania** | 39.37 (39.11 – 39.62) |
| **Wisconsin** | 38.95 (38.55 – 39.35) |
| **Virginia** | 38.18 (37.82 – 38.54) |
| **Idaho** | 36.88 (36.08 – 37.67) |
| **Illinois** | 36.79 (36.52 – 37.06) |
| **Wyoming** | 36.25 (34.93 – 37.56) |
| **New Jersey** | 36.04 (35.72 – 36.35) |
| **Colorado** | 34.95 (34.49 – 35.40) |
| **Nevada** | 33.88 (33.28 – 34.48) |
| **Florida** | 33.40 (33.21 – 33.59) |
| **New Hampshire** | 33.36 (32.58 – 34.13) |
| **Alaska** | 33.33 (31.85 – 34.82) |
| **Arizona** | 32.66 (32.31 – 33.02) |
| **Kansas** | 32.11 (31.59 – 32.63) |
| **Montana** | 31.28 (30.43 – 32.12) |
| **Maine** | 29.58 (28.90 – 30.27) |
| **Connecticut** | 27.02 (26.61 – 27.43) |
| **Utah** | 26.78 (26.19 – 27.38) |
| **Massachusetts** | 24.12 (23.83 – 24.41) |

**Table S9.** Diabetes mellitus and hypertension-related AAMR per 100,000 stratified by state in the United States from 2000 to 2023.

| **Census Region** | **Year** | **Age-Adjusted Rate (95% CI)** |
| --- | --- | --- |
| **Census Region 1: Northeast** | **2000** | 29.67 (29.12 – 30.23) |
| **Census Region 1: Northeast** | **2001** | 30.52 (29.97 – 31.08) |
| **Census Region 1: Northeast** | **2002** | 31.94 (31.38 – 32.51) |
| **Census Region 1: Northeast** | **2003** | 32.84 (32.27 – 33.41) |
| **Census Region 1: Northeast** | **2004** | 34.48 (33.90 – 35.06) |
| **Census Region 1: Northeast** | **2005** | 35.67 (35.08 – 36.26) |
| **Census Region 1: Northeast** | **2006** | 35.26 (34.67 – 35.84) |
| **Census Region 1: Northeast** | **2007** | 36.15 (35.56 – 36.74) |
| **Census Region 1: Northeast** | **2008** | 36.41 (35.82 – 36.99) |
| **Census Region 1: Northeast** | **2009** | 36.51 (35.93 – 37.10) |
| **Census Region 1: Northeast** | **2010** | 38.76 (38.16 – 39.36) |
| **Census Region 1: Northeast** | **2011** | 39.37 (38.77 – 39.97) |
| **Census Region 1: Northeast** | **2012** | 39.24 (38.65 – 39.83) |
| **Census Region 1: Northeast** | **2013** | 39.65 (39.06 – 40.24) |
| **Census Region 1: Northeast** | **2014** | 38.79 (38.20 – 39.37) |
| **Census Region 1: Northeast** | **2015** | 39.60 (39.01 – 40.18) |
| **Census Region 1: Northeast** | **2016** | 39.59 (39.01 – 40.18) |
| **Census Region 1: Northeast** | **2017** | 39.71 (39.13 – 40.28) |
| **Census Region 1: Northeast** | **2018** | 41.38 (40.79 – 41.96) |
| **Census Region 1: Northeast** | **2019** | 41.34 (40.76 – 41.92) |
| **Census Region 1: Northeast** | **2020** | 61.48 (60.77 – 62.18) |
| **Census Region 1: Northeast** | **2021** | 54.50 (53.84 – 55.16) |
| **Census Region 1: Northeast** | **2022** | 52.41 (51.78 – 53.05) |
| **Census Region 1: Northeast** | **2023** | 48.09 (47.49 – 48.70) |
| **Total** |  | 39.72 (39.13 – 40.32) |
| **Census Region 2: Midwest** | **2000** | 33.40 (32.85 – 33.95) |
| **Census Region 2: Midwest** | **2001** | 34.61 (34.06 – 35.17) |
| **Census Region 2: Midwest** | **2002** | 36.24 (35.67 – 36.80) |
| **Census Region 2: Midwest** | **2003** | 38.09 (37.52 – 38.67) |
| **Census Region 2: Midwest** | **2004** | 39.48 (38.90 – 40.07) |
| **Census Region 2: Midwest** | **2005** | 41.62 (41.02 – 42.21) |
| **Census Region 2: Midwest** | **2006** | 41.70 (41.11 – 42.29) |
| **Census Region 2: Midwest** | **2007** | 44.16 (43.56 – 44.77) |
| **Census Region 2: Midwest** | **2008** | 45.31 (44.70 – 45.92) |
| **Census Region 2: Midwest** | **2009** | 44.48 (43.88 – 45.08) |
| **Census Region 2: Midwest** | **2010** | 44.50 (43.91 – 45.10) |
| **Census Region 2: Midwest** | **2011** | 45.98 (45.38 – 46.58) |
| **Census Region 2: Midwest** | **2012** | 46.37 (45.77 – 46.97) |
| **Census Region 2: Midwest** | **2013** | 46.03 (45.44 – 46.62) |
| **Census Region 2: Midwest** | **2014** | 46.38 (45.79 – 46.97) |
| **Census Region 2: Midwest** | **2015** | 47.70 (47.11 – 48.30) |
| **Census Region 2: Midwest** | **2016** | 47.45 (46.86 – 48.05) |
| **Census Region 2: Midwest** | **2017** | 49.29 (48.69 – 49.89) |
| **Census Region 2: Midwest** | **2018** | 49.45 (48.86 – 50.04) |
| **Census Region 2: Midwest** | **2019** | 50.73 (50.14 – 51.33) |
| **Census Region 2: Midwest** | **2020** | 71.56 (70.86 – 72.26) |
| **Census Region 2: Midwest** | **2021** | 72.60 (71.88 – 73.32) |
| **Census Region 2: Midwest** | **2022** | 66.67 (66.00 – 67.35) |
| **Census Region 2: Midwest** | **2023** | 60.57 (59.93 – 61.21) |
| **Total** |  | 47.68 (47.08 – 48.29) |
| **Census Region 3: South** | **2000** | 36.26 (35.79 – 36.73) |
| **Census Region 3: South** | **2001** | 38.57 (38.09 – 39.05) |
| **Census Region 3: South** | **2002** | 40.99 (40.50 – 41.48) |
| **Census Region 3: South** | **2003** | 43.44 (42.94 – 43.94) |
| **Census Region 3: South** | **2004** | 43.68 (43.18 – 44.18) |
| **Census Region 3: South** | **2005** | 46.16 (45.66 – 46.67) |
| **Census Region 3: South** | **2006** | 46.83 (46.33 – 47.34) |
| **Census Region 3: South** | **2007** | 47.92 (47.42 – 48.43) |
| **Census Region 3: South** | **2008** | 48.31 (47.80 – 48.81) |
| **Census Region 3: South** | **2009** | 48.80 (48.30 – 49.29) |
| **Census Region 3: South** | **2010** | 49.89 (49.39 – 50.39) |
| **Census Region 3: South** | **2011** | 50.56 (50.06 – 51.05) |
| **Census Region 3: South** | **2012** | 51.61 (51.11 – 52.10) |
| **Census Region 3: South** | **2013** | 53.05 (52.55 – 53.54) |
| **Census Region 3: South** | **2014** | 52.18 (51.69 – 52.66) |
| **Census Region 3: South** | **2015** | 52.93 (52.45 – 53.41) |
| **Census Region 3: South** | **2016** | 54.89 (54.40 – 55.37) |
| **Census Region 3: South** | **2017** | 57.20 (56.72 – 57.69) |
| **Census Region 3: South** | **2018** | 58.77 (58.29 – 59.26) |
| **Census Region 3: South** | **2019** | 60.46 (59.97 – 60.95) |
| **Census Region 3: South** | **2020** | 84.13 (83.56 – 84.70) |
| **Census Region 3: South** | **2021** | 92.66 (92.06 – 93.27) |
| **Census Region 3: South** | **2022** | 84.51 (83.94 – 85.08) |
| **Census Region 3: South** | **2023** | 79.74 (79.19 – 80.29) |
| **Total** |  | 55.15 (54.64 – 55.65) |
| **Census Region 4: West** | **2000** | 33.61 (33.01 – 34.21) |
| **Census Region 4: West** | **2001** | 35.51 (34.90 – 36.12) |
| **Census Region 4: West** | **2002** | 37.96 (37.34 – 38.59) |
| **Census Region 4: West** | **2003** | 38.92 (38.30 – 39.55) |
| **Census Region 4: West** | **2004** | 40.77 (40.13 – 41.40) |
| **Census Region 4: West** | **2005** | 43.33 (42.68 – 43.97) |
| **Census Region 4: West** | **2006** | 44.42 (43.77 – 45.06) |
| **Census Region 4: West** | **2007** | 44.47 (43.83 – 45.11) |
| **Census Region 4: West** | **2008** | 45.73 (45.09 – 46.37) |
| **Census Region 4: West** | **2009** | 43.33 (42.72 – 43.95) |
| **Census Region 4: West** | **2010** | 45.45 (44.82 – 46.07) |
| **Census Region 4: West** | **2011** | 46.60 (45.98 – 47.22) |
| **Census Region 4: West** | **2012** | 46.64 (46.02 – 47.25) |
| **Census Region 4: West** | **2013** | 47.90 (47.29 – 48.52) |
| **Census Region 4: West** | **2014** | 46.64 (46.05 – 47.24) |
| **Census Region 4: West** | **2015** | 47.52 (46.93 – 48.11) |
| **Census Region 4: West** | **2016** | 48.61 (48.02 – 49.20) |
| **Census Region 4: West** | **2017** | 50.98 (50.38 – 51.58) |
| **Census Region 4: West** | **2018** | 50.85 (50.26 – 51.44) |
| **Census Region 4: West** | **2019** | 52.17 (51.58 – 52.76) |
| **Census Region 4: West** | **2020** | 68.81 (68.14 – 69.47) |
| **Census Region 4: West** | **2021** | 77.45 (76.73 – 78.17) |
| **Census Region 4: West** | **2022** | 68.85 (68.19 – 69.51) |
| **Census Region 4: West** | **2023** | 63.60 (62.97 – 64.23) |
| **Total** |  | 49.56 (48.93 – 50.18) |

**Table S10.** Diabetes mellitus and hypertension-related AAMR per 100,000 stratified by census region in the United States from 2000 to 2023.


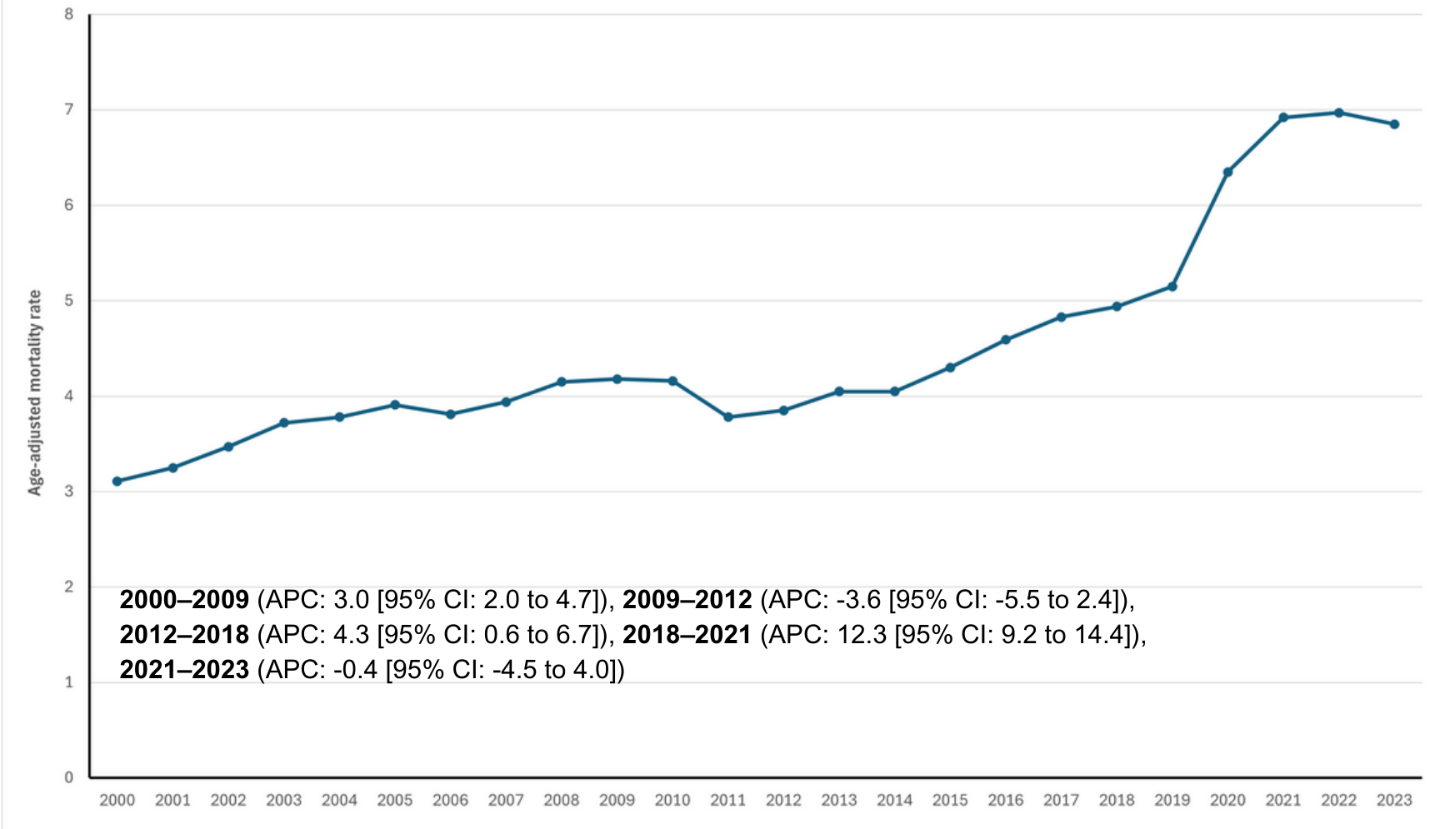


**Figure S1.** Hypertension-related AAMR per 100,000 in adults aged 25 years and older in the United States from 2000-2023.


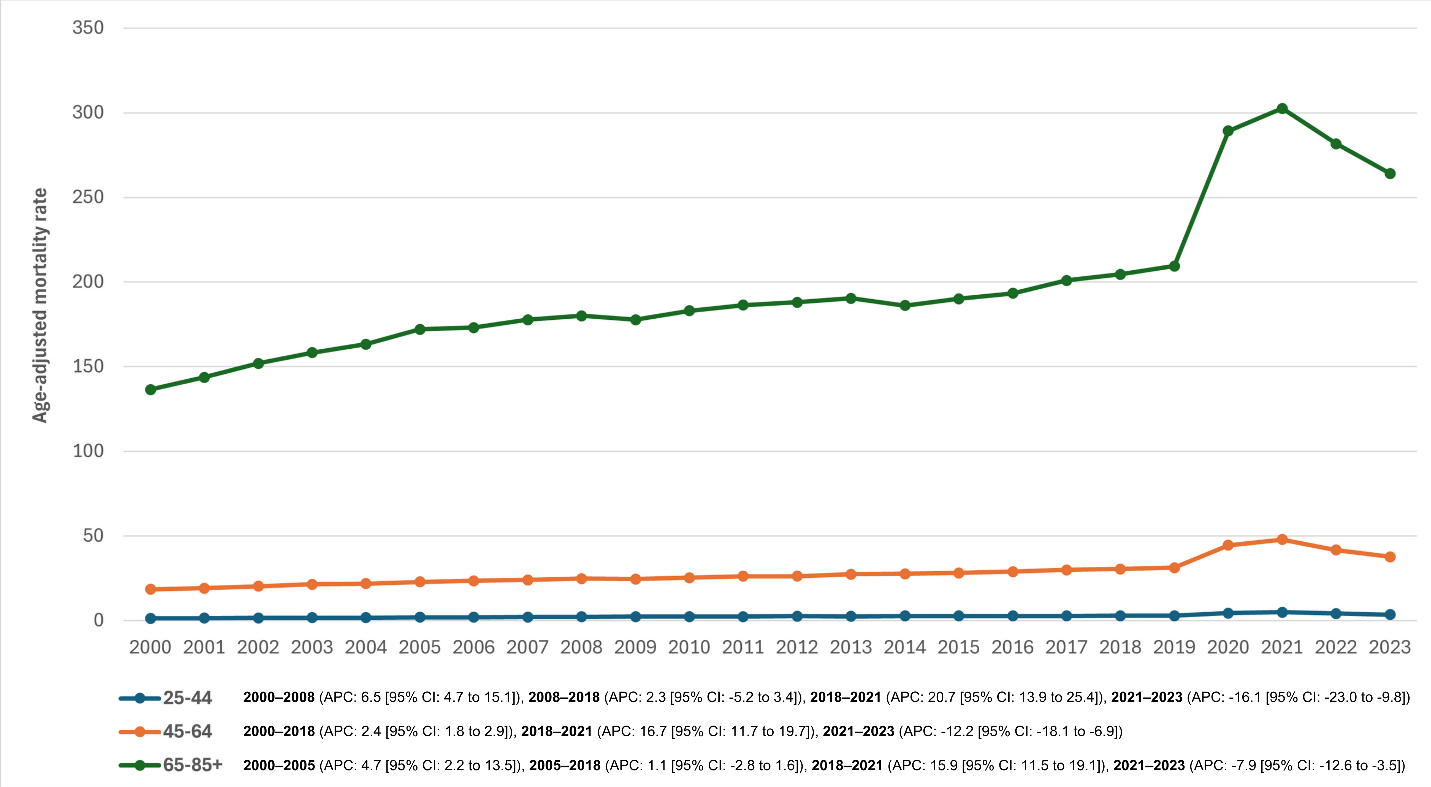


**Figure S2.** Trends in diabetes mellitus and hypertension-related mortality per 100,000 stratified by age in the United States from 2000 to 2023.


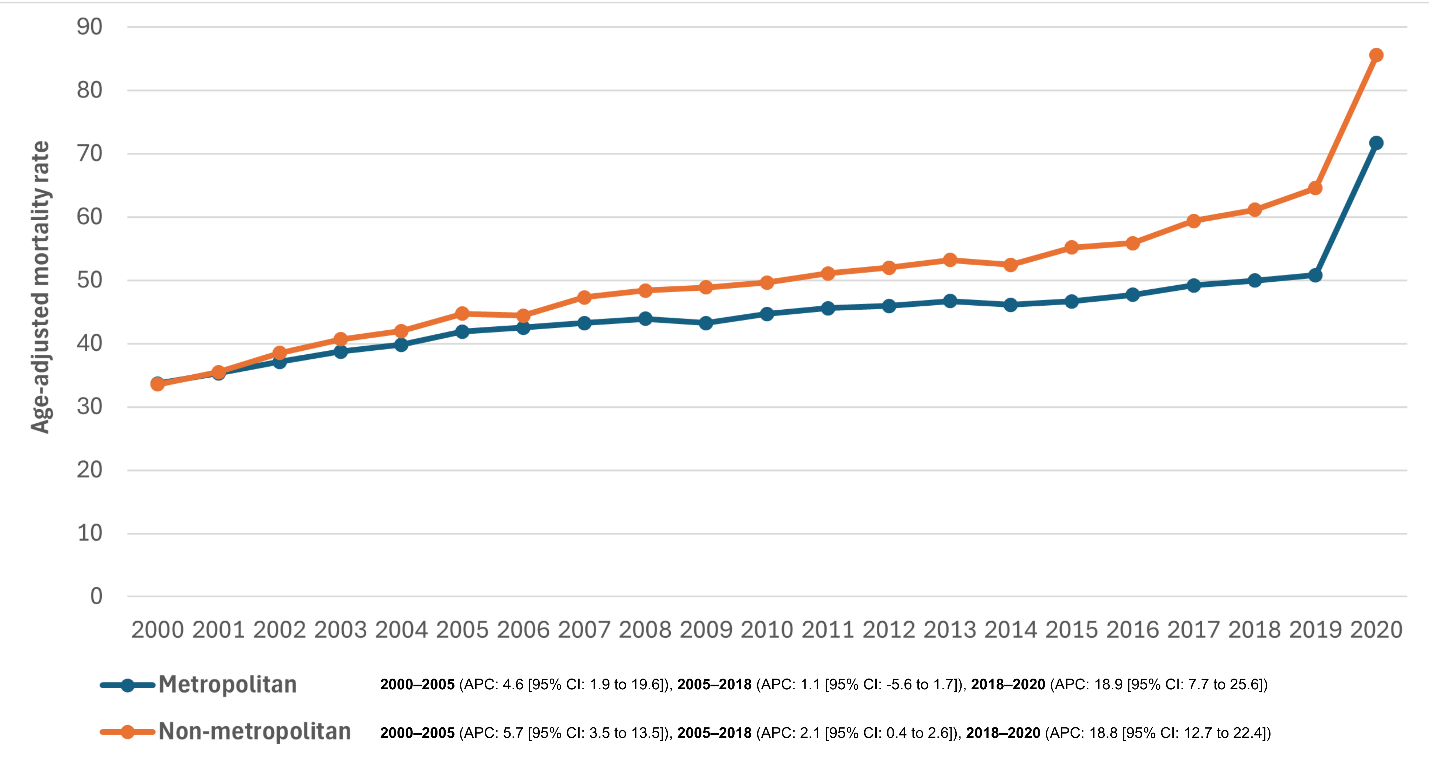


**Figure S3.** Trends in diabetes mellitus and hypertension-related mortality per 100,000 stratified by the level of urbanization in the United States from 2000 to 2023.
